# Neonatal thrombocytopenia: Thrombin generation in presence of reduced platelet counts and effects of rFVIIa in cord blood

**DOI:** 10.1038/s41598-019-44199-y

**Published:** 2019-05-29

**Authors:** Harald Haidl, Sina Pohl, Bettina Leschnik, Siegfried Gallistl, Wolfgang Muntean, Axel Schlagenhauf

**Affiliations:** Department of Pediatrics and Adolescent Medicine, Medical University of Graz, Graz, Austria

**Keywords:** Paediatric research, Preclinical research

## Abstract

Healthy neonates exhibit a well-functioning haemostatic system despite peculiarities regarding composition of clotting factors and inhibitors as well as impaired platelet aggregation. Thrombocytopenia and severe bleeding events are feared in sick infants. Recombinant factor VIIa (rFVIIa) is a haemostatic agent used as a last resort in neonates with refractory bleedings. Aim of this study was to investigate *in-vitro* (i) changes in thrombin generation with different platelet counts, (ii) effects of rFVIIa under conditions of thrombocytopenia and (iii) potentially differing dose-response of rFVIIa in cord blood as a surrogate for neonatal blood compared to adult blood. Thrombin generation parameters were observed in cord blood plasma and adult plasma with various platelet counts, with or without addition of rFVIIa, respectively. Low platelet counts did not influence thrombin generation in cord blood in contrast to adult blood. RFVIIa primarily affected lag time throughout all platelet concentrations. Interestingly, peak height was reduced exclusively in cord blood plasma after addition of rFVIIa. No significant differences regarding dose-response were observed between cord blood and adult blood. In contrast to adult blood, thrombocytopenia in cord blood does not significantly influence thrombin generation. Even at very low platelet counts there is enough negatively charged surface to support rFVIIa action in plasma from cord blood and adult blood *in-vitro*.

## Introduction

Healthy infants exhibit a well-functioning haemostatic system *in-vivo* and are not prone to easy bruising. The plasmatic coagulation system shows some particularities as many coagulation factors are known to be low at time of birth and adapt to adult levels within the first months of life. As shown by Cvirn *et al*., low procoagulatory factors are also accompanied by low levels of inhibitory factors, which result in a well-balanced haemostasis^1^.

Platelets of newborns exhibit impaired platelet function in *in-vitro* aggregation measurements^2^. This hypoaggregability is not due to a refractory state caused by preactivation during birth^3^. Multifactorial impairments in signal transduction have been shown to cause this hypoaggregability, including impaired calcium mobilization, lower numbers of α2-adrenergic receptors and lower GTPase activity in G_q_-coupled receptors^4–6^. Despite these impairments, the phospholipid composition of neonatal platelet membranes and the overall phospholipid surface expression upon activation are similar to that of adult platelets.

Thrombin generation is a pivotal step in the formation of a stable fibrin clot and highly dependent on negatively charged phospholipids. Neonatal platelets support thrombin generation equally to adult platelets due to the aforementioned comparable phospholipid composition^7^.

While healthy infants do not tend to bleed, preterm infants and sick infants exhibit a high bleeding risk, particularly when associated with thrombocytopenia which exacerbates the fragility of their haemostatic system. Thrombocytopenia with a threshold in platelet counts below 150,000/µl is observed in up to 35% of children that have to be treated – for any reason – at a neonatal intensive care unit (NICU) and is even more frequent and severe in preterm babies^8^. In cases of bleeding and/or platelet counts below 20,000/µl, platelet concentrates are given to increase platelet counts. In cases of refractory bleeding, recombinant factor VIIa (rFVIIa) is a therapeutic option^9^. However, fragility of the neonatal haemostatic system also leads to an increased risk of thrombosis^10^.

RFVIIa is licensed for haemophiliac patients with inhibitors, Glanzmann thrombasthenia and FVII deficiency, but it is also used in several off-label indications to cease bleeding that cannot be stopped otherwise. Successful administration in neonates and preterm babies^11–13^ as well as in older children^14^ is described for cases of refractory bleeding events with/without thrombocytopenia. On the other hand, the prophylactic administration of rFVIIa to preterm infants does not prevent intracranial bleeding^15^. A study about off-label uses of rFVIIa in childhood reports 3655 off-label administrations of rFVIIa; 39.8% in the first year of life, among these 48% within the first month of life. According to this study, venous and/or arterial thrombosis – the main adverse events – occurred in 10.9% of all pediatric off-label administrations (vs. 2.7% of all label administrations). In neonates the incidence rate was 13.6%^9^.

Mechanism of action of rFVIIa on platelets is a tissue factor-independent activation of FX in presence of phospholipids. Small amounts of thrombin are generated, leading to a thrombin burst^16^. The hypothesised mechanism of rFVIIa action in a thrombocytopenic state is a boost of the initial thrombin generation through high-dose rFVIIa, resulting in faster platelet activation, thus compensating for the lower number of platelets^17^.

Calibrated automated thrombography is an *in-vitro* method enabling time-dependent tracing of thrombin generation in various conditions. It has been shown that this method is applicable to detect states of hypo- as well as hypercoagulability. It is sensitive to clotting factor deficiencies and reflects the effect of anticoagulant drug^18–20^. When performed in the presence of platelet rich plasma the necessary phospholipids are provided by activated platelets. Therefore, this assay is superior to conventional global tests of haemostasis without platelets which are less sensitive and only detect onset of fibrin clot formation.

Considering aforementioned peculiarities of the neonatal haemostatic system we posed the question whether rFVIIa will perform in a similar manner in samples from adults and from cord blood with neonatal platelets featuring exactly those peculiarities. Particularly we wanted to elucidate the following issues: How does thrombin generation differ in samples from cord blood (CB) compared to adult samples when platelet counts are reduced and no rFVIIa is added? Is there a different *in-vitro* dose-response to rFVIIa in thrombin generation of platelet rich plasma (PRP) from CB compared to adult PRP, and does a lower dose eventually have a sufficient effect on thrombin generation? Does reduction of platelets influence the rFVIIa support of thrombin generation?

## Results

**Figure 1:**
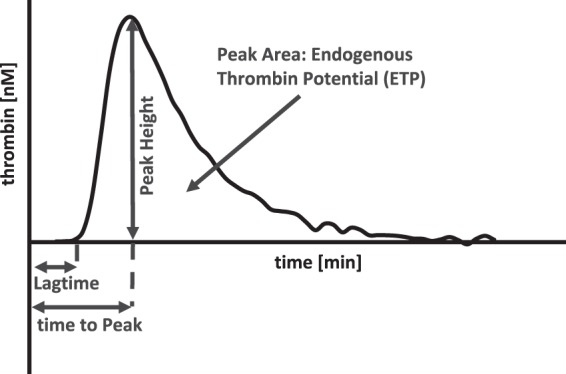
Schematic depiction of a thrombin generation trace. Endogenous thrombin potential (ETP) – area under the curve, represents the total amount of generated thrombin; lag time – time from tissue factor addition to onset of thrombin generation; peak – maximum level of generated thrombin; time-to-peak (ttpeak) – time from tissue factor addition to peak generation.

First, we evaluated potential differences between thrombin generation of platelet-rich plasma from cord blood and from adult blood. Figure 1 shows schematics of a thrombin generation trace with the corresponding parameters. In our study samples from cord blood exhibit a shorter lag time and time-to-peak and a higher peak. The endogenous thrombin potential (ETP, Table 1), however, is lower, which is similar to previous reports with platelet-poor plasma^1^. Taken together thrombin generation in platelet-rich plasma from cord blood differs from that of adult blood.

**Table 1:** Impact of 100,000 platelets/µl vs. 10,000 platelets/µl on thrombin generation parameters of all studied cord blood derived samples (n = 10) and adult samples (n = 10) without rFVIIa.

cord blood	100,000/μl	10,000/μl
lag time [min]	3.4 (±0.46)^§§^	3.5 (±0.60)^§§^
ETP [nMol × min]	1238.7 (±176.3)^§§^	1225.4 (±144.2)^§§^
peak [nMol]	168.1 (±27.8)^§^	168.9 (±27.3)^§§^
time-to-peak [min]	6.9 (±0.64)^§§^	7.1 (±0.74)^§§^
velix [nMol/min]	48.9 (±11.9)^§§^	49.0 (±14.1)^§§^
**adult blood**		
lag time [min]	4.6 (±0.56)	4.6 (±0.48)
ETP [nMol × min]	2106.3 (±208.1)	1860.3 (±144.6)**
peak [nMol]	144.7 (±27.6)	100.4 (±38.8)**
time-to-peak [min]	13.5 (±3.34)	11.7 (±2.69)
velix [nMol/min]	19.6.4 (±12.0)	17.3 (±11.1)

**Marks a significant difference between the two platelet counts (**p < 0.001). ^§^Marks a significant difference between adult and cord blood samples at the same platelet count (^§^p < 0.05; ^§§^p < 0.001). ETP (endogenous thrombin potential), velix (velocity index).

**Figure 2:**
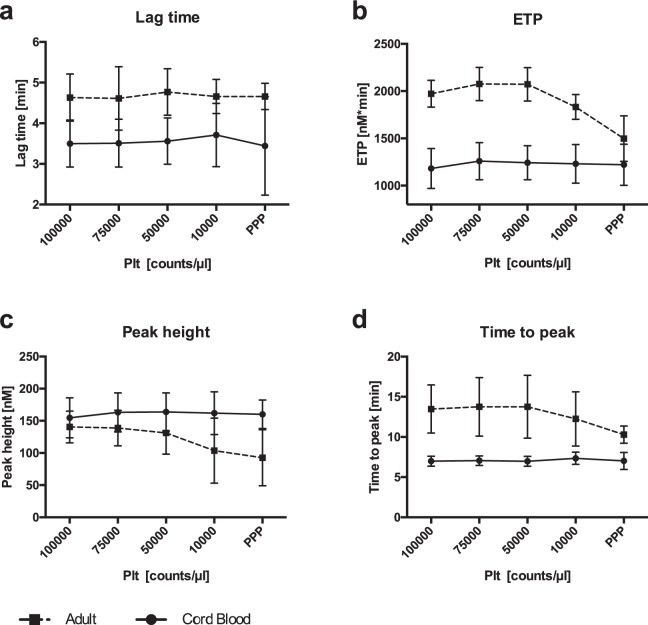
Impact of decreasing platelet counts on thrombin generation parameters without rFVIIa. Course of parameters of thrombin generation – lag time (**a**), endogenous thrombin potential (ETP) (**b**), peak height (**c**), time-to-peak (ttpeak) (**d**) – in cord blood derived and adult plasma with platelets at different platelet counts (100,000/µl, 75,000/µl, 50,000/µl, 10,000/µl and PPP; n = 5).

Next, we tested the impact of varying platelet counts on thrombin generation in samples from cord blood and adult blood. Table 1 shows effects of platelets on thrombin generation and parameters without rFVIIa at 100,000 and 10,000 platelets/µl (mean ± SD). While CAT parameters in cord blood derived plasma are comparable at platelet counts of 100.000/µl and 10.000/µl, the values for ETP and peak in adult plasma are significantly reduced at 10,000 platelets/µl compared to 100,000 platelets/µl. Parameters at 100,000, 75,000, 50,000, 10,000 platelets/µl and platelet poor plasma (PPP) in a subset of adult and cord blood (CB) samples are shown in Fig. 2. Values remain constant in CB samples whereas ETP and peak levels are reduced at 10,000 platelets/µl and PPP in adult samples. Taken together, thrombin generation in samples from adult blood exhibits a substantially higher platelet dependency than samples from cord blood.

Then, we tested the impact of rFVIIa on thrombin generation at varying platelet counts in samples from cord blood and from adult blood. The impact of rFVIIa on thrombin generation is primarily reflected in the shortening of lag time. Results of thrombin generation experiments – run with 1.5 µg/ml rFVIIa at different platelet concentrations – are outlined in Table 2 and are given as ratios ± SD to vehicle. The use of 3.0 µg/ml rFVIIa did not further increase the effects observed with 1.5 µg/ml rFVIIa (data not shown). The ratios provide comparability between CB and adult blood as absolute values differ broadly. For lag time shortening, the ratios reflect a more pronounced impact of rFVIIa in CB than in adult blood (Table 2) whereas the absolute reductions in lag time are within a similar range in CB and in adult blood (1.22–1.38 min and 0.91–1.13 min, respectively). Concerning peak heights the addition of rFVIIa leads to a reduction in CB but not in adult blood. Lower maximum amount of generated thrombin with rFVIIa might be due to lower prothrombin levels in CB leading to substrate consumption caused by accelerated initial thrombin generation rates. ETP shows no significant change, neither in the adult group nor in the CB group. These effects are comparable for all platelet concentrations. Taken together, we found a similar impact of rFVIIa in samples from cord blood and from adult blood, and no platelet-dependency in rFVIIa-action.

**Table 2:** Relative impact of decreasing platelet counts on all parameters of thrombin generation with 1.5 µg/ml rFVIIa vs. vehicle.

*cord blood*	lag time *[min]*	ETP	peak	ttpeak	velix
		*[nMol* × *min]*	*[nMol]*	*[min]*	*[nMol/min]*
100,000/µl	0.62 ± 0.02**^§^	0.96 ± 0.13	0.94 ± 0.04*	0.84 ± 0.03*^§^	0.87 ± 0.04*§
75,000/µl	0.65 ± 0.03*^§§^	0.93 ± 0.17	0.92 ± 0.05*	0.87 ± 0.05*^§^	0.85 ± 0.07*§
50,000/µl	0.65 ± 0.02**^§^	0.95 ± 0.12	0.91 ± 0.05*	0.86 ± 0.01**^§^	0.85 ± 0.07*
10,000/µl	0.66 ± 0.07*^§^	0.98 ± 0.02	0.93 ± 0.03*	0.87 ± 0.03*^§^	0.86 ± 0.10
PPP	0.64 ± 0.05*^§^	0.95 ± 0.10	0.91 ± 0.04*^§^	0.86 ± 0.03*^§^	0.82 ± 0.08*
***adult blood***					
100,000/µl	0.75 ± 0.06**	0.99 ± 0.03	0.98 ± 0.05	0.90 ± 0.04*	0.99 ± 0.09
75,000/µl	0.80 ± 0.03**	1.01 ± 0.06	0.99 ± 0.06	0.94 ± 0.03*	0.98 ± 0.08
50,000/µl	0.77 ± 0.05**	0.97 ± 0.03	0.95 ± 0.03	0.97 ± 0.08	0.89 ± 0.10
10,000/µl	0.76 ± 0.06*	0.98 ± 0.09	0.99 ± 0.07	0.93 ± 0.05*	0.94 ± 0.07
PPP	0.77 ± 0.03**	0.97 ± 0.05	0.96 ± 0.04	0.94 ± 0.04*	0.89 ± 0.08

Ratios for each parameter were formed from absolute values with/without rFVIIa. Samples from cord blood derived and adult plasma with platelet counts of 100,000/µl, 75,000/µl, 50,000/µl, 10,000/µl and platelet poor plasma (PPP) with rFVIIa at 1.5 µg/ml (n = 5). *Marks a significant difference to baseline level of the same platelet count (*p < 0.05; **p < 0.001). ^§^Marks a significant difference between adult and cord blood samples (^§^p < 0.05; ^§§^p < 0.001). ETP (endogenous thrombin potential), ttpeak (time-to-peak), velix (velocity index).

**Figure 3:**
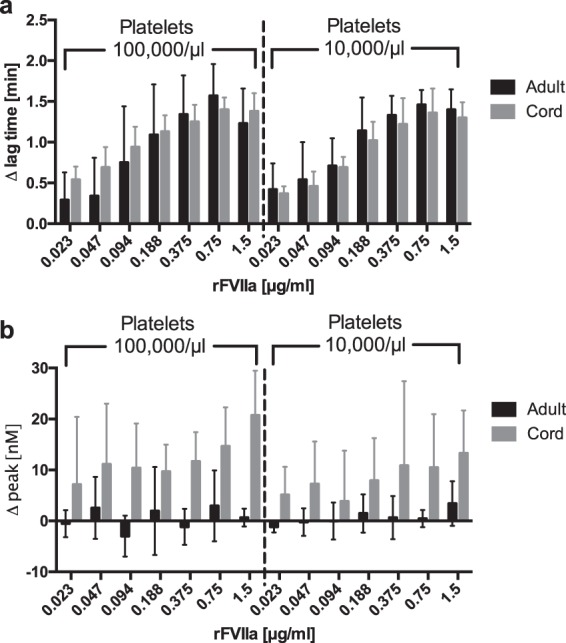
Impact of varying rFVIIa concentrations on reduction of lag time and peak thrombin generation. Lag time shortening (**a**) and changes in peak (**b**) were observed with different concentrations of rFVIIa at 100,000 platelets/µl and 10,000 platelets/µl (n = 5).

Finally, we evaluated potential differences in rFVIIa dose response of thrombin generation in samples from cord blood and from adult blood. Dose response of lag time shortening was analysed by performing thrombin generation experiments with different rFVIIa concentrations in PRP samples from CB and adult blood. Figure 3 shows results of experiments with PRP at 100,000 and 10,000 platelets/µl with varying rFVIIa concentrations compared to vehicle. In these tests, standard deviations are relatively high, reflecting the varying dose response of our samples. Significant differences in lag time reduction between CB and adult blood were not found. It is remarkable that even with the lowest concentration of rFVIIa (0.023 µg/ml), a measurable reaction could be observed in both groups. While ETP remains unchanged in these experiments, peak levels in CB samples abate gradually with higher rFVIIa levels (Fig. 3b). Taken together, overall dose response is comparable in samples from cord blood and adult blood, and both cohorts exhibit high sensitivity to rFVIIa even at very low concentrations.

**Figure 4:**
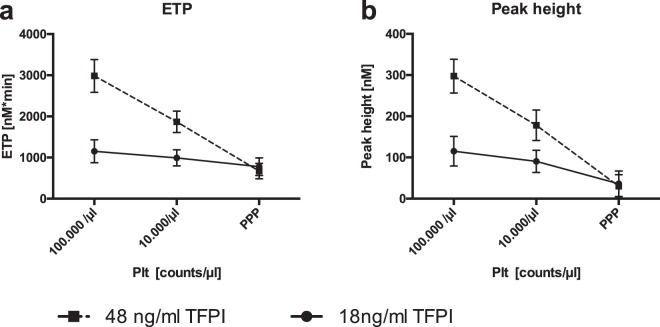
Impact of varying TFPI concentrations on platelet count dependency of thrombin generation. CAT measurements were done with TFPI depleted standard plasma reconstituted with recombinant TFPI (18 and 48 ng/ml). ETP (**a**) and peak (**b**) are depicted with varying counts of washed platelets added to the plasma samples (100,000/µl, 10,000/µl, PPP).

To explain the varying dependency of thrombin generation on platelet counts in samples from cord blood and adult blood, we performed additional experiments and evaluated the impact of differing TFPI-levels in standard plasma. TFPI depleted standard plasma reconstituted to neonatal (18 ng/ml) or adult (48 ng/ml) TFPI levels show similar platelet-dependencies as plasma from CB and adult blood respectively (Fig. 4). Samples with 48 ng/ml TFPI exhibit a substantial reduction in ETP and peak while samples with 18 ng/ml TFPI show only minor changes with decreasing platelet counts. Taken together, thrombin generation in samples with lower TFPI levels is less dependent on the presence of platelets.

## Discussion

Aim of our study was to evaluate the support of thrombin generation by neonatal platelets and the effect of factor rFVIIa simulating thrombocytopenia *in-vitro*. Neonatal platelets show impaired aggregation *in-vitro* but no apparent lack in function *in-vivo* since primary haemostasis seems to be functional in healthy newborns^21–23^. Specific peculiarities of neonatal platelets explain this hypoaggregability^24–26^. We wanted to study the role of platelet counts in thrombin generation in cord blood (CB) and the effects of procoagulant agent rFVIIa under these circumstances.

In our thrombin generation experiments with adult samples and no rFVIIa, low platelet counts of 10,000/µl lead to diminished endogenous thrombin potential- (ETP-) and peak-levels compared to 100,000/µl, while lag time and time-to-peak were not influenced by platelet counts (Fig. 2). In CB samples no remarkable change was observed at declining platelet numbers in any of the thrombin generation parameters.

Apparently, normal thrombin generation can be established in CB, even with low platelet counts. In our experimental setup the source of phospholipids, which are required to support thrombin generation, are endogenously derived from platelets.

There is no activation of platelets during birth and the phospholipid content in CB does not differ from adults^3,7^. Possibly, lower amounts of negatively charged phospholipids are required to optimally support thrombin generation in newborns due to lower plasma levels of tissue factor pathway inhibitor (TFPI) and antithrombin (AT) in the neonatal haemostatic system. Additionally, elevated microparticles in CB may contribute to the overall phospholipid content to provide sufficient support of thrombin generation^27^. We tested this hypothesis by mimicking neonatal and adult TFPI levels using TFPI depleted standard plasma and recombinant TFPI. Our results showed that ETP and peak are less dependent on platelet counts at lower TFPI levels hinting to a substantial role of TFPI in platelet dependency of thrombin generation. Interestingly, ETP and peak were higher with 48 ng/ml than with 18 ng/ml TFPI when ample amounts of platelet surface were available (100.000/µl). This may seem contradictory to TFPI’s inhibiting function but fits well with thrombin generation observed in plasma from CB and adult blood. Possibly, TFPI plays an additional role in the positive feedback loop of thrombin generation by thrombin itself.

Our findings differ from Gerotziafas *et al*., who described an elongation of lag time and time-to-peak in adult samples with low platelet counts. Presumably, these contrasts are due to unknown tissue factor concentrations and the addition of exogenous phospholipids in their study^28^.

Using rFVIIa, we observed a similar dose response in CB derived plasma and in adult samples. The total amount of generated thrombin (ETP) shows no significant change after adding rFVIIa, but the kinetics of reaction change. The effect of rFVIIa lies in the acceleration of the initial thrombin burst, resulting in shortened lag time and time-to-peak. The shortening of lag time after addition of rFVIIa in newborn (and preterm) platelet poor plasma has already been shown by Streif *et al*. with a subsampling method^29^. These findings are now also observed in thrombocytopenic CB derived plasma and adult plasma, respectively. We did not find significant differences of rFVIIa-effects at varying platelet counts. In our experiments, the mean percentage of shortening was 62% to 66% of negative control in CB plasma and 75% to 80% in adult plasma, not depending on platelet counts. We conclude that a reduction of platelets does not influence the rFVIIa support of thrombin generation.

Interestingly, the absolute values for lag time shortening were similar for adult and cord blood samples. This is noteworthy because – without rFVIIa – lag time in samples from CB was already shorter than in adult samples. We postulate that plasmatic coagulation factors, especially the lower levels of tissue factor pathway inhibitor (TFPI), contribute to a comparable shift despite lower absolute baseline lag time in CB samples, which resulted in a higher relative reduction than in adult samples^1^. Reduction of peak levels with rFVIIa was only observed in samples from cord blood. Possibly, lower TFPI levels and low amounts of prothrombin in neonates result in a higher fraction of prothrombin being converted by rFVIIa. Thus, less prothrombin might be remaining for peak thrombin generation via FVIII/FIX.

The presented results from *in-vitro* experiments were done with 1.5 μg/ml rFVIIa, which roughly corresponds to 90 μg/kg, the recommended dose in a clinical setting. Additionally, we tried several lower concentrations as we were interested in dose dependent effects on thrombin generation in cord plasma, taking into consideration that physiological levels of FVII are lower in newborns. Therefore, we would have expected a lower dose of rFVIIa to reach the same effect when compared to adults. Administered rFVIIa doses in neonates vary greatly within the literature. In a case series of 36 newborns, the dose of rFVIIa was mostly received with good response but varied broadly between 15 µg/kg and 200 µg/kg^30^. Greisen *et al*. describe the effect of rFVIIa-doses of 5 µg/kg and 80 µg/kg in preterm babies with prolonged prothrombin time (PT). PT was already significantly shortened at the lowest dose (5 µg/kg)^31^. Usually, a supraphysiological dose of rFVIIa is needed for pharmacological purposes. As shown by Shibeko *et al*., this need can not only be explained by a phospholipid-related pathway but also by a competition between rFVIIa and factor VII zymogen (FVII) for tissue factor binding^32^.

Our results do not support this assumption as we did not find a maximum lag time shortening at levels below 1.5 μg/ml, neither at 100,000 nor at 10,000 platelets/µl. Therefore, in our experiments we found no evidence for a heightened dose response in neonates and comparable saturating rFVIIa concentrations.

Limitations of such trials lie in the use of cord blood, which may not reflect a neonatal *in-vivo* situation ideally. It is, however, the only ethically justifiable way to achieve sufficient quantities of neonatal platelets. We presumed a platelet count of 100,000/µl to be functionally normal as usually no bleeding occurs and higher levels were not feasible in the long term. Presentation of data as ratios was done to establish comparability of study cohorts, but may marginally distort the results as small changes at lower baseline levels affect ratios more than at higher baseline levels.

In summary, we found that even at low platelet counts exhaustive rFVIIa action is observable in plasma from cord blood as well as from adult blood. The *in-vitro* hypoaggregability of neonatal platelets does not result in an impaired support of thrombin generation with or without rFVIIa. Nevertheless, the neonatal haemostatic system is known to be fragile. Peculiarities of neonatal haemostasis should be taken into consideration in addition to the critical health state, when rFVIIa has to be administered to babies due to the relatively high thrombosis rate in this age cohort.

## Methods

This study was approved by the local ethics committee (Ethikkommission der Medizinischen Universität Graz), and all participants gave written informed consent in accordance with the Declaration of Helsinki.

Each experiment was repeated five-times with a total of ten different participants. Blood of adult volunteers (5 males, 5 females) was drawn with a 21 gauge needle from the antecubital vein, without applying venostasis, into precitrated S-Monovette premarked tubes (3 ml) from Sarstedt (Nümbrecht, Germany), containing 0.30 ml 0.106 mol/l trisodium citrate solution. Cord blood was obtained from term newborns (gestational age 38–42 weeks), immediately following delivery into the abovementioned citrate tube and processed shortly. For thrombin generation experiments, platelet-rich plasma (PRP) was obtained by centrifugation (200 × g, 10 min, 20 °C) and platelet count was measured using a Sysmex KX 21 cell counter. Platelet-poor plasma (PPP) was obtained by centrifugation (1600 × g, 10 min, 20 °C).

RFVIIa (Eptacog alpha; Novoseven^©^, Novo Nordisk A/S, Danmark) was reconstituted according to manufacturer’s instruction and stored in aliquots of 35 µl at −80 °C for later use. These were diluted with NaCl 0.9% to obtain the desired concentration.

### Thrombin generation experiments

PRP was diluted with autologous platelet poor plasma (PPP) to produce platelet counts of 100,000, 75,000, 50,000 and 10,000/µl. Thrombin generation was performed using Calibrated Automated Thrombography according to Hemker *et al*. as reported previously^20,27^. This method allows the continuous tracing of thrombin generated *in-vitro* in a specific plasma sample over time. Thrombin generation assays were performed using a Fluoroskan Ascent plate reader (Thermo Labsystems, Helsinki, Finland) and Thrombinoscope^©^ software (Thrombinoscope BV, Maastricht, the Netherlands). 20 µl of PRP-reagent (1 pM tissue factor final concentration) or calibrator (both Thrombinoscope BV, Maastricht, The Netherlands) and rFVIIa in a final concentration of 1.5 µg/ml and 3.0 µg/ml were placed into respective wells of a 96-well-plate (Immulon 2 HB, Thermo Scientific). 80 µl PRP with the respective platelet counts were added. The measurement was started by automatic dispensing of 20 µl fluobuffer-CaCl_2_ (20 mM Hepes, 140 mM NaCl, 5 mg/ml bovine serum albumin, and 16.7 mM CaCl_2_ final concentration, pH 7.35), containing a fluorogenic substrate (Z-Gly-Gly-Arg-amino-methyl-coumarin, Bachem, Bubendorf, 417 µM final concentration). Thrombin generation profiles were recorded in triplicates. Further thrombin generation experiments were performed with platelet counts of 100,000 and 10,000/µl (+platelet poor plasma, PPP) and varying concentrations of rFVIIa (1.5, 0.75, 0.38, 0.19, 0.09, 0.047, 0.023 µg/ml). The thrombin generation parameters lag time, endogenous thrombin potential (ETP), peak, time-to-peak (ttpeak) and velocity index (velix) were calculated from respective profiles. Figure 1 shows schematics of a thrombin generation trace with the corresponding parameters. The velix was calculated with the formula [peak thrombin/(peak time − lag time)].

In a follow-up experiment, TFPI-depleted plasma (Sekisui Diagnostics, Lexington, MA) was reconstituted with recombinant TFPI (18 ng/ml or 48 ng/ml, Abcam, Cambridge, UK) to mimic neonatal or adult TFPI levels. Washed platelets from an adult donor were added to a subset of these plasma samples with platelet counts of 100,000 and 10,000/µl, respectively, before CAT measurements.

### Statistical analysis

Data are presented as mean ± SD or relative changes ± SD. Differences within cord blood or adult samples were analysed using ANOVA. Corrections for multiple comparisons were made using the Holm-Šídák method (α = 0.05), and multiplicity adjusted P-values were calculated for each comparison. Graphpad Prism 6.0 (Graphpad software, San Diego, CA) was used for performing calculations and creating figures. The datasets generated and/or analysed during the current study are available from the corresponding author on request.

### Ethical approval

All procedures performed in studies involving human participants were in accordance with the ethical standards of the institutional and/or national research committee and with the 1964 Helsinki declaration and its later amendments or comparable ethical standards.
